# Reply to Editorial in the *Dental Cosmos*

**Published:** 1894-08

**Authors:** Chas. N. Taft

**Affiliations:** Chicago, Ill.


### Domestic Correspondence.

### REPLY TO EDITORIAL IN THE "DENTAL COSMOS."

To the Editor:

Sir,—There appears in the April issue of the *Dental Cosmos* a most excellent editorial under the caption "Dental Scientific Work," and it may seem strange that any one could find reasonable ground for passing adverse criticism upon it, or for questioning the sincerity of the thoughts to which the editor has so ably given expression.

It is probably true that the great majority of dental practitioners have neither the time, the means, nor the inclination to engage in original research or study which would add one iota to the common fund of knowledge in any single department of our calling, and that such work is, as the editorial admits, confined to a small proportion of workers who, imbued with the love of finding out the truth for the truth's sake, are ever working, each along different lines and by different methods, to find out some new thing which can be given to the whole profession for the benefit of mankind.

Work of this character, when entered upon in the proper spirit, is, unquestionably, "a labor of love which brings its full compensation in the results achieved," and rarely, if ever, involves any self-sacrifice; but I doubt very much if there was ever a man in our ranks who began faithfully and conscientiously the work of scientific investigation with no baser purpose in mind than to give the results of such investigations freely and cheerfully to his brother practitioners who did not meet with abundant opposition of some kind, or who failed to have the satisfaction, sooner or later, of knowing that such work had proved to be of help to others, or the means of encouraging them to still greater achievements along a similar line of work.

And yet it is strange, but nevertheless true, that there are journals, or rather, shall I say, a *single* journal, claiming upon its title-page to be "devoted to the interests of the profession," whose columns it is given out to be interpreted, editorially, are always open for the publication of papers reporting the results of original work, and yet declining at times to publish such papers when offered.

"Only comparatively rarely," does the editorial article assert, "are reports or statements made which are founded upon original research or experiment, or even upon accurately-observed individual phenomena." If it is true, as the editor assures us in the following lines, "that it is only such matter that can possess any scientific importance or lasting value," he does not make it quite clear just *why* "it must be evident that we are wasting time and energy in giving it attention."

If we are to credit the accuracy and sincerity of the first part of this statement, with whom lies the fault if such reports, when sent to the journals, are not given the widest possible publicity?

Again, if it is true, as the editorial goes on to say,—and I have no reason to dispute the assertion,—"that to properly observe and interpret selected phenomena is the initial step in scientific investigation, and the ability to correctly reason and generalize upon the results so obtained is what constitutes the scientific method of thought," it certainly cannot be out of place for me to say here that it is upon that very line that I have been conducting my own investigations during the last three or four years in the department of therapeutics, for the reason that this department offers to me at least a fascinating and increasingly interesting field of work, and one in whatever success I may have achieved has proved in its practical and clinical application especially gratifying in its results.

It is known to many that my work in this direction has been conducted always with the one single purpose of adhering strictly to the homœopathic law of similars as laid down by Samuel Hahnemann, and for the purpose of encouraging others *never* to accept the results of my work upon my own *ipse dixit*, but rather to first prove all things for themselves and hold fast only to that which is good.

It was this spirit which prompted me to prepare a paper entitled "The Philosophy of the Homœopathic Law of Cure, and the Advantage to the Dentist of a Correct Understanding of its Application," read before the Massachusetts Dental Society at its twenty-seventh annual meeting, and afterwards to submit it to the *Dental Cosmos*, that it might be "published broadcast for the benefit of my colleagues," but which, for some reason best known to the publishers, was returned to me with a letter stating that it was "not considered available for the purposes of that journal," whatever that phrase may mean. The paper was subsequently published in the November, 1892, issue of the INTERNATIONAL DENTAL JOURNAL.

It cannot be denied that the editorial is a timely one, and has the proper ring to it; and it most assuredly *ought* to inspire the young, the active, and the brainy men in our ranks to cultivate the spirit and love for scientific study, not alone for their own benefit, but for the good of the entire profession; but it seems to me a pertinent thing for some one to request of the *Dental Cosmos* an open definition of the phrase in its application to the acceptance or the rejection of papers that modestly claim to be scientific. It would furnish at least a common ground of understanding between editors and dentists as to what really constitutes dental scientific work, and what course must be pursued with the *Dental Cosmos*

from now on, before the results of such investigations can be published in the broadcast manner so ably advocated by its universally esteemed and respected editor.

It was Lord Bacon who once said, "I hold every man to be a debtor to his profession: from the which, as men do seek to receive honor and profit, so should they by way of amends endeavor to be a help and ornament thereto."

Aside from the fact that there is nothing in the editorial which at first sight would call forth anything but words of the highest praise and commendation for the sentiments expressed, there is, nevertheless, under the circumstances to which I have referred, a vein of grim humor running through it which only goes to prove the fact, or point the moral, if one there be, that there are journals and journals,—some that are unquestionably devoted to the interests of the *whole* profession, others that are only apparently so.

Yours truly,

Chas. N. Taft.

Chicago, Ill., May 5, 1894.

